# A Cross-Sectional Survey to Assess the Preparedness of Healthcare Workers of Isolation Intensive Care Units for Anticipated Another Wave of the COVID-19 Pandemic in a Tertiary Care Center

**DOI:** 10.7759/cureus.54949

**Published:** 2024-02-26

**Authors:** Annu Choudhary, Kumar Saurabh, Alok Kumar, Nidhi Arun

**Affiliations:** 1 Anesthesiology, Indira Gandhi Institute of Medical Sciences, Patna, IND; 2 Anesthesiology and Critical Care, Indira Gandhi Institute of Medical Sciences, Patna, IND

**Keywords:** cross-sectional study, covid-19, pandemic, intensive care unit, health personnel

## Abstract

Background: Our fight against coronavirus disease 2019 (COVID-19) has not ended yet. Another wave, possibly due to another variant, can put the healthcare system on its toes again. The outcome of the intensive care unit (ICU) depends on the teamwork of doctors, nursing staff, and care assistants (CAs).

Aim: This survey was conducted to assess the level of preparedness of healthcare workers (HCWs) in isolation ICUs by recording and calculating the score of their responses, using a validated pre-formed questionnaire.

Methods: A cross-sectional survey was conducted on 200 HCWs in isolation ICUs by using a pre-validated questionnaire form as an assessment tool. HCWs in isolation ICUs included doctors, nurses, and CAs who have worked in ICUs dedicated to COVID-19 patients. The response was documented and scores as per the response were assessed by analog scale.

Results: Most of the HCWs were anxious (53%) and 96.5% were either bothered or scared. HCWs with adequate knowledge have higher generalized anxiety disorder scores (χ2 = 15.287, p = 0.015). Despite having adequate/average knowledge, most of the HCWs have insufficient knowledge of the correct steps of donning and doffing.

Conclusion: HCWs were aware of the COVID-19 pandemic but were not prepared for the anticipated second wave.

## Introduction

We, the healthcare workers (HCWs) have faced, fought, and survived the coronavirus disease 2019 (COVID-19) pandemic [1]. Our fight against the pandemic has taught us many lessons. We now understand the importance of good physical and mental health, a healthy lifestyle, and a robust healthcare system. The vaccination drive conducted by the Government of India to promote herd immunity has given us hope [2]. Various strains of the COVID-19 virus have been isolated with a spectrum of severity of presentation [3]. Another wave, possibly due to other variants or strains, can put the healthcare system on its toes again. We must be better prepared, equipped, and aware to work in a coordinated way to perform effectively.

The outcome of the intensive care unit (ICU) depends on teamwork [4]. Better patient care and outcomes in the ICU are only possible if every HCW performs his responsibilities efficiently. Doctors are the leaders whereas nursing staff, physiotherapists, dieticians, and care assistants (CAs) like sanitation workers, hospital attendants, and patient transporters are other critical components of the ICU team. That is why, we need to assess their awareness, knowledge, and preparedness level individually, to offer them customized support.

The primary objective of this study was to assess the level of preparedness of HCWs in isolation ICUs by recording and calculating the score of their responses, using a validated pre-formed questionnaire.

## Materials and methods

This cross-sectional survey was conducted after ethical approval from the institutional ethics committee and registration with the Clinical Trial Registry, India [ctri.nic.in] vide registration number CTRI/2021/11/037802 at Indira Gandhi Institute of Medical Sciences, Patna, Bihar, India. A validated pre-formed questionnaire was used as the tool for assessment. ICU HCWs were defined as all the HCWs who have worked in the isolation ICU dedicated to patients with COVID-19. This included regular ICU HCWs and HCWs from other units who have joined to strengthen the ICU team during the pandemic. Each HCW was contacted and invited to participate in the study by filling out the questionnaire form. Participation in the study was on a voluntary basis and completely anonymous. Participants were required to provide their employee’s identification number to limit the number of responses to one per HCW. Incompletely filled responses in the questionnaire were screened and rejected by one investigator, and then the selected filled questionnaires were assessed for quality check by another investigator.

Assuming about 50% of the HCWs have good preparedness with a 95% confidence interval and 8% absolute error, we found the sample size to be 151. Considering an attrition rate of 20%, the final sample size calculated was 182. To round off, we selected a sample size of 200.

A questionnaire form was given to interested HCWs of isolation ICUs willing to participate in this study. HCWs in isolation ICUs included doctors, nurses, and CAs. The pre-formatted questionnaire form contained information on the purpose of the study and a set of questions with scores allotted to every answer. The response was documented, and scores as per the response were assessed by the analog scale.

Assessment tool

Our questionnaire included questions regarding demographic profile, and questions to assess their mental status, knowledge, and working experience in isolation ICUs. Some scoring systems used were well-known pre-validated scales like generalized anxiety disorder (GAD) and peri-traumatic distress inventory (PDI) scores and others like scoring systems for lifestyle modification, fear, inter-personal relationship, knowledge, performing donning and doffing in the correct order, fitness score and preparedness score were designed, revised, and validated, by us as per the results of a pilot study [5,6]. 

Our questionnaire contained questions under three sections (Appendix 1). The first section consisted of eight questions focused on the participant’s general information. The extent of lifestyle modification was assessed by five different questions.

The second section was designed to assess the mental status of HCWs while working in isolation ICU. It included a GAD scale to assess anxiety which contains seven questions to establish the severity of anxiety. A set of four questions was prepared to assess fear. PDI is a pre-validated score to assess peri-traumatic distress (PTSD) through 13 questions regarding the participant’s emotional and physiological distress experienced during and immediately after a traumatic event. The COVID-19 pandemic and witnessing closely the catastrophe caused by this, while working in isolation ICUs was defined as the traumatic event in this study. PDI more than or equal to 14 predicted PTSD one month after the traumatic event. Question each was asked to describe their working experience in isolation ICU and to know how their interpersonal relationship with co-workers was affected.

The third section was prepared to assess the knowledge and experience of HCWs. It included a set of 20 basic questions related to COVID-19 infection and management with 3 one-word closed response options. We also tried to know their response regarding the usefulness of training programs. Knowledge of the correct order of donning and doffing was assessed by asking HCWs to re-arrange the steps of donning and doffing in the correct order. For every correct step, 1 point was given and the total score was the sum of all points.

Based on the results of the pilot study, we concluded that the level of preparation of HCWs was independently influenced by their knowledge and mental health. So, using three parameters 1) Fitness score, 2) GAD score, and 3) PDI score, we developed the preparedness score. The fitness score was the result of adding the scores for the right donning and doffing sequence, understanding of the COVID-19 infection, fear of working in an isolation ICU, and lifestyle modification. Mental status was assessed by GAD and PDI scores. Fitness score ≥ 47, GAD score ≤ 9, and PDI score ≤ 13 were considered as cut-off values for preparedness score.

We piloted the initial survey in English among 30 HCWs and revised it accordingly. We then translated the instrument into Hindi language. We reverse-translated the Hindi version, pre-tested it, and amended the final text as necessary. 

The data were recorded and analysis was done using IBM SPSS Statistics for Windows, Version 27 (Released 2020; IBM Corp., Armonk, New York, United States). The qualitative data were shown in terms of percentages. The quantitative data were expressed either in percentages or in mean and standard deviations. The Chi-square or Fisher exact test was used to analyze the differences between the two proportions. Student t-test was used to find out the difference between the two means. Non-parametric tests were used for the analysis of data that were not normally distributed. The point-biserial correlation coefficient was derived to assess the strength of association. The threshold for statistical significance was p < 0.05.

## Results

**Figure 1:**
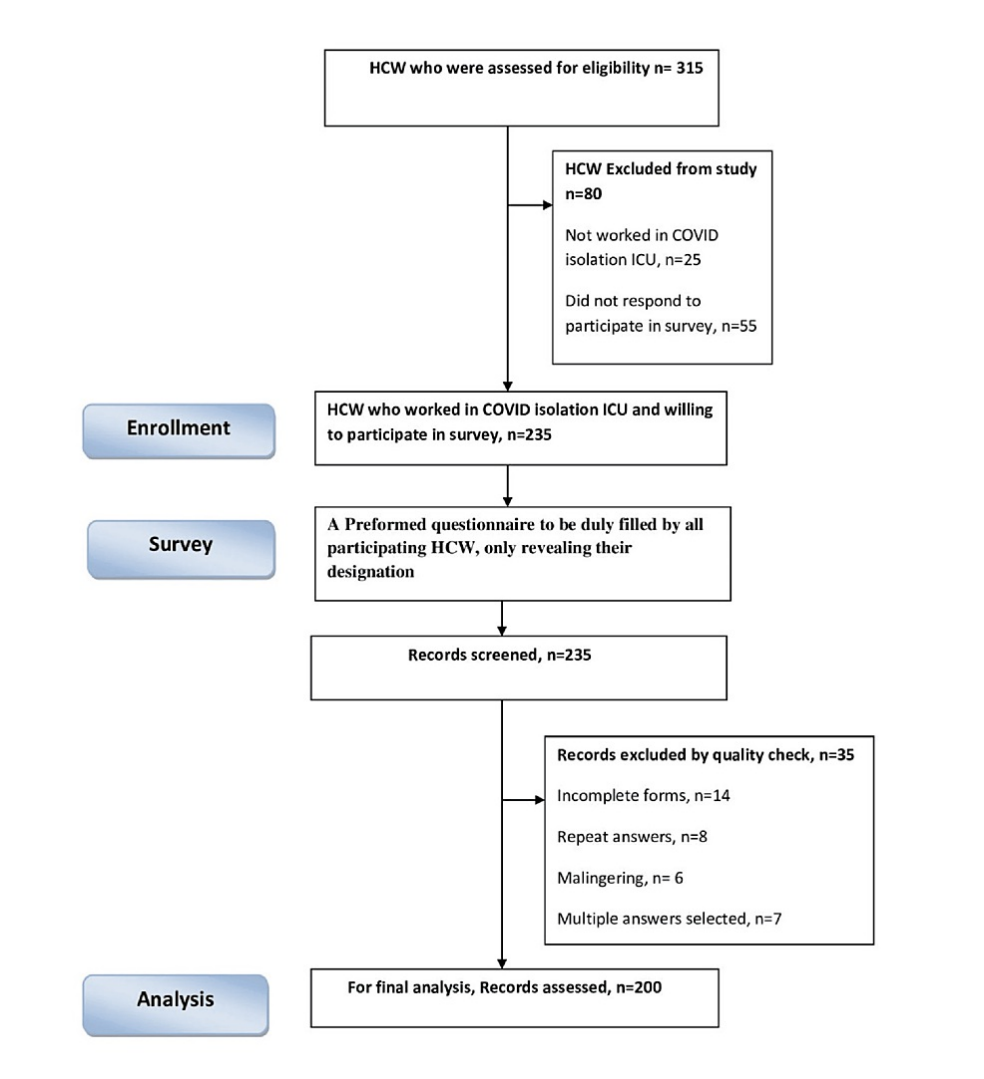
Strengthening the reporting of observational studies in epidemiology (STROBE) diagram HCW: Healthcare worker; ICU: intensive care unit

Two hundred and thirty-five responses from HCWs of isolation ICUs were received. After quality check, 200 responses were selected for final analysis (Figure 1).

A survey was conducted on 200 HCWs which included 100 doctors (50%), 50 nurses (25%), and 50 CAs (25%). The demographic parameters are shown in Table 1. 

**Table 1:** Demographic parameters SD: Standard deviation; CAs: care assistants

S. No.	Parameter	Doctor (n=100)	Nurses (n=50)	CAs (n=50)	Total (n=200)
1.	Age in years (Mean ± SD)	33.35±5.09	28.16±.39	31.22±5.73	31.53±5.33
2.	Male (%): Female (%)	53:47	48:52	82:18	59:41
3.	Married (%): Unmarried (%)	63:37	54:46	88:12	67:33
4.	Regular (%): contractual (%)	85:15	20:80	6:94	49:51

**Figure 2:**
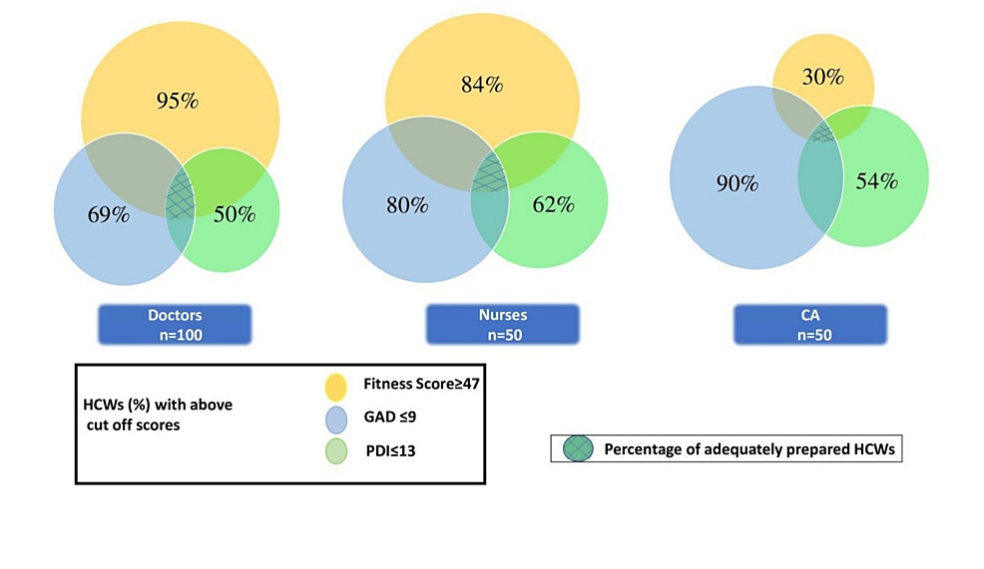
Three circles represent the percentage of HCWs with the above cut-off score in three components – i) Fitness score, ii) GAD score, and iii) PDI score, respectively. The common shaded area represents the percentage of adequately prepared HCWs among doctors, nurses, and CAs HCWs: Healthcare workers; GAD: generalized anxiety disorder; PDI: peri-traumatic distress inventory; CAs: care assistants

Based on the preparedness score, 34% of doctors, 42% of nursing staff, and 18% of CAs were found to be prepared for anticipated another wave of COVID-19 (Figure 2).

Forty-six percent of HCWs reported signs and symptoms of PDI. No co-relation was found between COVID-19 infection and PDI among HCWs (p = 0.760). Most of the HCWs who were anxious had signs and symptoms of PDI. Fifty-three percent of HCWs had anxiety, out of which 66 were male and 40 were female. No significant difference between the various groups in terms of distribution of gender was seen (χ2 = 5.823, p = 0.121HCWs) with no anxiety had a stronger interpersonal relationship with other HCWs (Bias Corrected Cramer's V = 0.2). Other associations are depicted in Table 2. 

**Table 2:** Association between the GAD scale and fear level, working experience, and knowledge GAD: Generalized anxiety disorder

		GAD scale						Fisher’s exact test	
S. No	Parameter		None	Mild	Moderate	Severe	Total	X^2^	P value
1.	Fear level								
a.	No fear		6(6.5%)	0(0%)	1(3%)	0(0%)	7(3.5%)	9.176	0.185
b.	Bothered		36(38.7%)	20(33.9%)	8(24.2%)	3(21.4%)	67(33.7%)		
c.	Scared		51(54.8%)	39(66.1%)	39(66.1%)	11(78.6%)	125(62.8%)		
2.	Level of knowledge								
a.	Adequate		55(59.8%)	30(51.7%)	28(84.8%)	12(85.7%)	125(63.5%)	15.287	0.015
b.	Average		33(35.9%)	27(46.6%)	5(15.2%)	2(14.3%)	67(34.0%)		
c.	Little		4(4.3%)	1(1.7%)	0(0%)	0(0%)	5(2.5%)		
3.	Working experience								
a.	Satisfying		8(8.6%)	1(1.7%)	1(3.0%)	0(0%)	10(5%)	10.714	0.113
b.	Hectic		63(67.7%)	33(55.9%)	18(54.5%)	9(64.3%)	123(61.8%)		
c.	Traumatic		22(23.7%)	25(42.4%)	14(42.4%)	5(35.7%)	66(33.2%)		

On the assessment of the fear level, we observed that 33.5% of HCWs were bothered and 63% were scared of COVID-19. Association of fear with different factors was assessed (Table 3).

**Table 3:** Association of the fear level with marital status, type of service, vaccination status, working experience, and level of knowledge

Fear level						Fisher’s exact test	
S. No.	Parameter	No Fear	Bothered	Scared	Total	X^2^	P-value
1.	Marital Status						
a.	Married	3(42.9%)	46(68.7%)	85(67.5%)	134(67%)	1.941	0.405
b.	Unmarried	4(57.1%)	21(31.3%)	41(32.5%)	66(33%)		
2.	Type of service						
a.	Regular	0(0%)	23(34.3%)	75(59.5%)	98(49%)	18.081	<0.001
b.	Contractual	7(100%)	44(65.7%)	51(40.5%)	102(51%)		
3.	Vaccination status						
a.	Single dose	2(28.6%)	9(13.4%)	4(3.2%)	15(7.5%)	12.991	0.006
b.	Double dose	5(71.4%)	56(83.6%)	121(96%)	182(91%)		
c.	None	0 (0%)	2(3%)	1(0.8%)	3(1.5%)		
4.	Working experience						
a.	Satisfying	1(14.3%)	9(13.4%)	0(0%)	10(5%)	23.437	<0.001
b.	Hectic	6(85.7%)	42(62.7%)	76(60.3%)	124(62%)		
c.	Traumatic	0(0%)	16(23.9%)	50(39.7%)	66(33%)		
5.	Level of knowledge						
a.	Adequate	3(42.9%)	31(47%)	92(73.6%)	126(63.6%)	15.298	0.002
b.	Average	4(57.1%)	32(48.55)	31(24.8%)	67(33.8%)		
c.	Little	0(0%)	3(4.5%)	2(1.6%)	5(2.5%)		

We observed that most of the HCWs had arranged the doffing sequence in the correct order while failing to do the same for the donning sequence. We analyzed the association of level of knowledge with awareness of the correct order of donning and doffing sequence separately in doctors, nurses, and CAs as they belong to different educational backgrounds. So, their aptitude and way of perceiving things were expected to be different (Table 4).

**Table 4:** Association of the level of knowledge with adequate knowledge of donning and doffing sequence among HCWs (n=200) HCWs: Healthcare workers; CAs: care assistants

Knowledge													
S. No.	Parameter	Adequate					Average			Little			P-value
1.	Donning sequence (%)	Doctor	Nurses	CA			Doctor	Nurses	CA	Doctor	Nurses	CA	>0.05
a.	Correct	24.7	5.9	0			16.7	0	15	0	0	25	
b.	Incorrect	70	94.1	0			83.3	16	85	0	0	75	
2.	Doffing sequence												
a.	Correct	81.9	41.4		0	83.3		46.2	48.7	0	75	0	<0.05
b.	Incorrect	18.1	58.6		0	16.7		53.8	51.3	0	25	0	

## Discussion

A prepared HCW has adequate knowledge along with mental agility to bear the stress of the pandemic. We found that only 32% of HCWs in our center were prepared to face the anticipated wave of the pandemic. On further analysis, we found that most of the doctors and nursing staff had adequate knowledge but were less mentally prepared. On the other hand, CAs lacked both knowledge and mental strength.

The two domains that were taken into consideration to gauge the preparedness of HCWs were knowledge and mental health. Both of these domains are equally important and distinct from one another. We therefore included the fitness score, GAD, and PDI score to evaluate total preparedness. This would guide us in determining the shortcomings in various domains in different categories of HCWs and enable us to plan the lacunae-focused training modules. This will prepare them to deal with immense stress inside the isolated ICU and treat patients effectively without compromising their health [7].

To the best of our knowledge, no studies have been conducted on hospital attendants, patient transporters, sanitation workers, etc. However, we considered them as an essential part of the ICU team. Their insight and responsiveness are equally important. So, the response of such 50 team members was included in our survey to have a comprehensive outlook of the whole ICU team.

We found that 53% of HCWs had anxiety and 45.5% reported PDI. We did not find any significant difference in the level of anxiety based on gender. However, a survey conducted by other authors has observed that female HCWs experienced more negative emotional, physical, and behavioral changes than male HCWs. This gender disparity was not related to knowledge of HCWs [8]. We observed the signs and symptoms of PDI in HCWs who were anxious. During the pandemic, the whole world was blown out with an exponential number of cases that crumpled our existing healthcare system. Our country was under lockdown and people were too fearful to meet their friends and family. These had an overall traumatic impact on the mental status of HCWs. Despite having adequate knowledge, training, and resources, our HCWs became victims of depression and post-traumatic stress disorder. Jang et al. conducted an online survey in South Korea to find burnout and peri-traumatic distress in HCWs during the COVID-19 pandemic. They reported that PTSD was inversely proportional to the adequacy of training, knowledge, and optimum rest between working shifts in isolation ICU [9]. Contrary to this, we found that the level of anxiety was directly proportional to the level of knowledge. We speculate that more awareness regarding the grave prognosis of the disease, uncertainty of definite treatment, scarcity of resources, and disproportionately high disease burden induced anxiety among HCWs. They also found that HCWs working under high pressure in uncertain conditions had negative effects on their social and mental health contributing to exhaustion. HCWs with lower GAD scores reported better interpersonal relationships with other HCWs in their team. Teo et al conducted a multi-centric online survey on HCWs to assess stress, anxiety, and burnout during the COVID-19 pandemic in Singapore and reported that teamwork and being appreciated at work are the two most important factors associated with lesser anxiety. They suggested job dedication, emotional support and self-efficacy are other protective factors that could reduce stress levels in HCWs [10].

Fear is defined as an aversion response to a defined situation (COVID-19 pandemic). Anxiety and fear are co-related and can lead to PDI [11]. However, most of the HCWs in our survey were either bothered or scared, irrespective of their anxiety level. Though most of the regular HCWs had adequate knowledge and were vaccinated with both doses, they were either bothered or scared. The causes of fear are multiple, based on individual mental status, associated co-morbidities, family support, ability to handle stress, and attitude of the community toward them.

We observed that despite adequate knowledge, only a few HCWs arranged the doffing sequence correctly, despite having an adequate or average level of knowledge. This shows that HCWs were more careful and alert while doffing than donning. There was a time gap between the second wave of the pandemic and our survey. HCWs might have forgotten the correct order of donning and doffing during this period. We should emphasize in our training programs that donning and doffing are equally important and have their significance in providing protection. Contrary to this observation, the incidence of COVID-19 infection in these HCWs was quite less (37%). If they had not performed the donning and doffing steps correctly, we would have found a higher infection rate among them. They must have followed the correct order of donning and doffing while working in the isolation ICU, looking at the pictorial banners or charts displayed in the changing room without remembering them. More than half of HCWs (61.8%) appreciated their working experience as hectic. This might be due to long working hours, continuous use of personal protective kits (PPE) and high patient load [12]. Only 5% of HCWs felt working in isolation ICU was satisfying and 80% of them had no anxiety.

Garg et al. found in their knowledge, attitude and practice (KAP) survey published in 2020 that donning and doffing are cumbersome and lengthy procedures [13]. HCWs felt that the importance of this practice had been exaggerated. They found that HCWs understood that PPE is critical for personal safety but they also reported that this is inconvenient and this practice could not be continued for longer. These might be the reasons behind the non-compliance of HCWs toward correct donning and doffing steps.

We suggest reinforcement of the safety practices into the habit of HCWs by repeated training, testing, and motivating them to follow these protocols. Separate modules of training programs in the local language for different classes of HCWs should be prepared and implemented. Special attention should be given to promoting sound mental health of HCWs. Training programs must potentiate self-esteem and teamwork. Various activities to de-stress like yoga sessions, sports, cultural events etc. should be organized and HCWs should be encouraged to participate along with their family and friends. These training programs should be conducted at frequent intervals with feedback from the participants. We insist on monthly mock drills for donning and doffing in all HCWs. To encourage participation in training programs, incentives in any form may be introduced by the management authority.

Limitations

This is a single-center study with a relatively small sample size, conducted between January 2022 to June 2022, so results cannot be generalized to HCWs worldwide. We preferred printed questionnaires in English and Hindi over online forms as our study sample included HCWs of all categories. We tried to evaluate the level of anxiety, fear and PDI through a questionnaire but this cannot replace direct interviewing and clinical examinations. We have not asked about the history of any psychiatric disorders or their history of stress, anxiety or job satisfaction. This might also affect the attitude of HCWs during a pandemic. There was a gap of a few months between the duty period in the isolation ICU and the conduct of this survey. So, recall bias is probable in responses. This was a cross-sectional survey conducted at a single time point. We recommend regular follow-up surveys with updated questions to assess the knowledge, attitude, and practice of HCWs.

## Conclusions

We noticed that our HCWs were not prepared for a new pandemic. Most of the HCWs fell short in the domain of mental health. Contrarily, CAs lagged in both knowledge and mental health domains. This type of timely survey should be conducted to find the gaps in the ICU working system and thus modify it according to the need, deficit, and situation.

## Appendices

Questionnaire

1.      Category of HCW:   a) Doctor

                                           b) Nurse + paramedical + physiotherapist

                                           c) Sanitation staff + hospital attendant + patient transporter

2.      Age/Sex:

3.      Marital status:

4.      Total no. of family members:

5.      Type of service:    Regular/Contractual

6.      Have you ever contracted Covid-19 infection while working in ICU

7.      Vaccination status: No / single dose / two doses; If Not vaccinated, specify the reason…………………….

8.      Any lifestyle modification

**Table 5:** Assessment of lifestyle modifications Results will be interpreted as follows – Score 5 -8 = No lifestyle modification, 9-11 =Little lifestyle modification and 12-15 = Significant lifestyle modification

S. no.	Lifestyle modification	1= less than usual	2=same as usual	3=more than usual
1	Healthy eating habits			
2	Exercise habits			
3	Sleeping habits			
		1 = more than usual	2 = same as usual	3 =less than usual
4	Smoking habits			
5	Alcohol consumption			

      9.  Assessment of mental status

A] Generalized Anxiety Disorder (GAD) scale will be used to assess anxiety. It uses 7 questions to establish the severity of anxiety and results are interpreted as follows: 0 to 4: no anxiety, 5 to 9: mild anxiety, 10 to 14: moderate anxiety, 15 to 21: severe anxiety [3]. In order to define the presence of symptoms of anxiety, we will use a cut-off score ≥ 5.

·       Over the last two weeks, how often have you been bothered by the following problems?

**Table 6:** Generalized Anxiety Disorder (GAD) scale

S.no	Symptoms	Not at all	Several days	More than half the days	Nearly every day
1	Feeling nervous, anxious or on edge	0	1	2	3
2	Not being able to stop or control worrying	0	1	2	3
3	Worrying too much about different things	0	1	2	3
4	Trouble relaxing	0	1	2	3
5	Being so restless that it is hard to sit still	0	1	2	3
6	Becoming easily annoyed or irritable	0	1	2	3
7	Feeling afraid as something awful might happen	0	1	2	3

Total Score = Add Columns

10.    For assessment of fear 

**Table 7:** Scale for fear assessment

S. no.	Fear factors	0= Scared	1 = Bothered	2 = No fear
1	Fear of getting self infected			
2	Fear of spread to near and dear			
3	Fear of post-Covid complications			
4	Fear that existing facilities will fall short of anticipated next wave			

Results will be interpreted as follows - Score 0 = Scared, 1-4 = Bothered, and 5-8 = No fear.

11. The PDI is a validated score that was designed to evaluate peri-traumatic distress (PTSD) through 13 questions regarding the participant's emotional and physiological distress experienced during and immediately after a traumatic event [4]. PDI ≥ 14 has been shown to predict PTSD one month after a traumatic event, which will be defined here as the COVID-19 pandemic.

**Table 8:** Peri-traumatic distress inventory (PDI)

S. no	Item description	0= not at all	1= slightly	2= some what	3= very	4= extremely true
1	I felt helpless to do more					
2	I felt sadness and grief					
3	I felt frustrated or angry I could not do more					
4	I felt afraid for my safety					
5	I felt guilt that more was not done					
6	I felt ashamed of my emotional reactions					
7	I felt worried about the safety of others					
8	I had the feeling I was about to lose control of my emotions					
9	I had difficulty controlling my bladder and bowel					
10	I was horrified by what happened					
11	I had physical reactions like sweating, shaking and pounding heart					
12	I felt I might pass out					
13	I thought I might die					

12. How will you define your experience of working in isolation ICU? 1 = Traumatic, 2 = Hectic,    

     3=Satisfying

13. Change in inter-personal relationship between HCW-  1= weakened, 2 = no change, 3=strengthened

14. Assessment of knowledge:

**Table 9:** Inventory for assessment of knowledge

S. No	Questions	0 = No	1 = Little	2 = Adequate
1	Do you know about the causative organism of COVID			
2	Do you know about the mode of spread of COVID			
3	Are you aware about COVID safe protocols			
		0= less important	1= important	2= very important
4	How important PPE kit is?			
5	Importance of proper seal of N95 mask			
6	How important is to maintain hand hygiene?			
7	Importance of proper disposal of contaminated bio-medical wastes?			
8	Importance of sanitization of surface and equipment?			
9	Importance of role assignment in isolation ICU			
10	Importance of team work			
11	Importance of communication with patient’s attendants			
12	Importance of psychological support to the patient as well as family			
13	Importance of awake proning in COVID patients			
14	Importance of basic ICU care and nutrition in COVID patients			
15	Severity of COVID in ICU patients	0 = Mild	1 = moderate	2 = severe
16	Outcome of Covid in ICU	0 = Treated	1= Treated with residual damage	2 = Death
17	Knowledge of oxygen delivering devices	0= No	1= Basic	2 = Advance
18	Knowledge of methods of non-invasive and invasive ventilation	0 = No	1= Basic	2= Advance
19	Do you know about sanitization methods of surface and equipment	0=No	1= Little	2=Adequate
20	Do you know how to position patient for prone ventilation	0 = No	1 = Perhaps	2 = Yes

Scores will be interpreted as; 0-10 = Least knowledge, 11-20 = Little knowledge, 21-30 = Average knowledge,

31-40 = Adequate knowledge

15. Assessment of knowledge of correct order of donning and doffing -

  i.         Arrange the following steps of donning in correct order-

1.      Wear fit N 95 mask and check leak

2.      Wear goggles, face-shield and 2nd pair of gloves which should cover free end of gown sleeves

3.      Gown fit check

4.      Wash hands and wear 1st pair of gloves

5.      Wear shoe cover

6.      Wear coverall after checking its integrity

Correct order: ………

For every correct step, 1 point will be given. Total points will be interpreted as; 0-4= incorrect and 4-6 = correct

  ii.         Arrange the following steps of doffing in correct order-

1.      Remove shoe cover, perform hand hygiene

2.      Remove outer gloves, perform hand hygiene

3.      Remove face shield or goggles and coverall, perform hand hygiene

4.      Disinfect hands while wearing gloves using alcohol hand rub

5.      Remove  inner gloves, wear new pair of gloves and remove N95 mask slowly

6.      Sit over clean chair, clean your shoes with alcohol swab

7.      Remove last pair of gloves and perform hand hygiene

Correct order:…………………..

For every correct step, 1 point will be given. Total points will be interpreted as; 0-5= incorrect and 6-7 = correct
